# Estimation of intraocular lens position from full crystalline lens geometry: towards a new generation of intraocular lens power calculation formulas

**DOI:** 10.1038/s41598-018-28272-6

**Published:** 2018-06-29

**Authors:** Eduardo Martinez-Enriquez, Pablo Pérez-Merino, Sonia Durán-Poveda, Ignacio Jiménez-Alfaro, Susana Marcos

**Affiliations:** 1 Instituto de Óptica “Daza de Valdés”, Consejo Superior de Investigaciones Científicas (CSIC), C/Serrano, 121, 28006 Madrid, Spain; 2 Fundación Jiménez Díaz, Madrid, Av. Reyes Católicos, 2, 28040 Madrid, Spain

## Abstract

In a cataract surgery, the opacified crystalline lens is replaced by an artificial intraocular lens (IOL). To optimize the visual quality after surgery, the intraocular lens to be implanted must be selected preoperatively for every individual patient. Different generations of formulas have been proposed for selecting the intraocular lens dioptric power as a function of its estimated postoperative position. However, very few formulas include crystalline lens information, in most cases only one-dimensional. The present study proposes a new formula to preoperatively estimate the postoperative IOL position (ELP) based on information of the 3-dimensional full shape of the crystalline lens, obtained from quantitative eye anterior segment optical coherence tomography imaging. Real patients were measured before and after cataract surgery (IOL implantation). The IOL position and the postoperative refraction estimation errors were calculated by subtracting the preoperative estimations from the actual values measured after surgery. The proposed ELP formula produced lower estimation errors for both parameters -ELP and refraction- than the predictions obtained with standard state-of-the-art methods, and opens new avenues to the development of new generation IOL power calculation formulas that improve refractive and visual outcomes.

## Introduction

Age-related cataract is a cause of blindness on a global scale (43% of worldwide blindness) due to biological aging, genetic, and environmental factors of the crystalline lens, which loses its transparency. Cataract surgery has become a routine surgical procedure, with a treatment rate of 22 million annual surgeries worldwide^1^. Typically, the opacified crystalline lens is removed and replaced by a foldable intraocular lens (IOL). To optimize the visual quality after surgery, it is crucial to individually select the optimal IOL power to be implanted^2^. In current practice, the IOL power is usually calculated by using statistical regression formulas obtained from a large population sample^3,4^ or, more accurately, using theoretical formulas^2,5–13^.

Deviations in the preoperative estimation of postoperative IOL position, i.e., the estimated lens position (ELP), represent the largest contribution to error in modern IOL power calculation formulas^2,13–15^. Therefore, improvements in the ELP will provide better IOL power selection and thus refractive and visual outcomes.

Different generations of theoretical formulas have been proposed since 1970s, which vary in the way in which they estimate the lens position (ELP): the first generation assumed a constant value for the ELP^5,7^; the second generation individualized the prediction by replacing the constant ELP by one variable dependent on the axial length (AL) measured for every patient^6,11^; the third generation formulas (i.e. SRK/T formula^9^, Holladay formula^8^, or the Hoffer Q formula^12^) used axial length and anterior corneal curvature to predict ELP. Fourth and fifth generations included the preoperative anterior chamber depth (ACD) to improve the prediction (Olsen^16^ and Haigis formulas^10^, respectively).

In all of these approaches, the ELP is estimated from parameters unrelated to the shape of the crystalline lens. Recent approaches included crystalline lens thickness (e.g., the Olsen formula^13^, or more recently, the Olsen *et al*. C-constant^17^) or 2-dimensional measurements of the central area of the crystalline lens (e.g., the so-called intersection approach^18^).

To our knowledge, 3-dimensional (3-D) quantitative estimations of the preoperative full crystalline lens geometry and position have never been used, in part due to the unavailability of full crystalline lens quantitative data. Nevertheless, it seems reasonable that the estimation of where the IOL will be placed within the capsular bag will largely benefit from accurate 3-D information of the crystalline lens geometry.

Optical coherence tomography (OCT) is an excellent technique to image the anterior segment of the eye due to its high-resolution and high-speed, and provided with fan and optical distortion correction algorithms^19,20^, allows an accurate 3-D quantification of the geometry of the anterior segment of the eye^21^ including the central part of the crystalline lens^22^. We have recently presented and validated a method to accurately estimate and quantify the full shape of the crystalline lens from OCT data visible through the eye’s natural pupil aperture^23^.

In this study we propose a new formula to estimate the ELP based on preoperative 3-D full crystalline lens parameters (lens volume, surface area, diameter and equatorial plane position) measured in pre-cataract surgery patients. We compare the predictions (estimated lens position and refractive error) of the new formula with those from the SRK/T formula, the Olsen’s C constant and the intersection approach.

To our knowledge, this is the first work reporting a formula that relates 3-D full shape of the crystalline lens parameters and ELP.

## Material and Methods

### Patients, Surgery and Clinical Measurements

Twelve eyes from seven patients (79 ± 5 years old) were measured before and after cataract surgery (2 to 4 months from surgery). All patients received Carl Zeiss CT Asphina 409 M (Zeiss, Jena, Germany) IOL. IOL power ranged from +19.50 D to +24 D. The SRK/T formula was used to calculate the IOL power, selecting the first myopic value closest to emmetropia. Preoperative refractive errors ranged between −2 to +3.25 D sphere and −2.25 to −0.5 D cylinder. The surgery was performed at Fundación Jiménez Díaz by a single surgeon (SD-P) using phacoemulsification through a 2-mm incision at 90°. A 6-mm continuous curvilinear capsulorhexis was made under viscoelastic material. Phacoemulsification of the lens was performed with a commercial microsurgical system (Stellaris Microsurgical System; Bausch & Lomb, Rochester, NY).

The study met the tenets of the Declaration of Helsinki. Ethical approval was granted by the Ethics Committee of Consejo Superior de Investigaciones Científicas (CSIC). Written informed consent was obtained from the patients after detailed explanation of the procedure.

Table 1 shows the patient’s clinical profile, including the preoperative axial length (AL) measured with IOLMaster (Carl Zeiss AG, Jena, Germany), the actual IOL power implanted for each patient, and the postoperative final objective refraction (spherical equivalent, SE) obtained from auto-refractometry (Topcon, Tokyo, Japan).

**Table 1:** Patients clinical data.

	Male/Female	Age (y.o)	Axial length (mm)	IOL power (D)	Postoperative objective refraction (SE) (D)	Clinical grade of Cataract (BCN10)
S#1 (OD)	Female	80	23.10	22.0	0.750	N5
S#2 (OS)	Female	82	22.37	22.5	−1.125	N5
S#3 (OD)	Female	87	22.15	23.5	0.375	N6
S#3 (OS)	Female	87	22.08	23.5	0.125	N6
S#4 (OD)	Female	74	22.41	22.0	−0.370	N5
S#4 (OS)	Female	74	22.37	22.0	−0.625	N5
S#5 (OD)	Male	78	22.71	22.0	0	N6
S#5 (OS)	Male	78	22.62	22.0	0.250	N5
S#6 (OD)	Male	75	23.75	19.5	−0.250	N5
S#6 (OS)	Male	75	23.83	19.5	−0.125	N5
S#7 (OD)	Female	78	23.22	24.0	0.250	N4
S#7 (OS)	Female	78	23.35	24.0	0.625	N4

D = diopters; IOL = intraocular lens; SE = spherical equivalent.

The A-constant used for the IOL power calculations was 118.3, based on manufacturer recommendations. Table 1 also shows the clinical grade of cataract following the grading system BCN 10^24^, which ranges between clear lens (N0) to fully opacified nuclear cataract (N10).

### Optical Coherence Tomography Imaging

A custom-developed spectral OCT system, described in detail in previous publications^20,25^, was used to acquire the OCT images. The light source is a superluminiscent diode (λ_0_ = 840 nm, ∆λ = 50 nm). The set-up is based on a fiber-optics Michelson interferometer configuration, and the detector is composed of a spectrometer and a CMOS camera. The effective acquisition speed is 25000 A-Scans/s and the axial range is 7 mm in depth in air, resulting in a theoretical pixel resolution of 3.4 µm. The axial resolution is 6.9 µm in tissue.

One 3-D volume was composed of 300 A-scans, and 50 B-scans on a 10 × 10 mm lateral area, acquired in 0.6 seconds, which led to a good trade-off between time acquisition and resolution. Measurements were acquired after inducing mydriasis with one drop of tropicamide. The OCT axis was aligned with the pupilary axis by asking the patient to follow a fixation stimulus until the iris appeared flat in the preview OCT horizontal and vertical cross-sections^21,26^.

The axial range is not sufficiently large to capture all anterior segment surfaces in a single acquisition. In the preoperative measurements three sets of 3-D images were captured sequentially 5 seconds after blinking: (1) cornea, (2) anterior lens and (3) posterior lens. In the postoperative measurements two sets were needed (1) cornea and (2) IOL, shifting axially the plane of focus; all 3-D sets of data contained the iris. At least seven repeated measurements were collected for each plane of focus. Not useful measurements (e.g., measurements with blinking) were discarded from the analysis.

**Figure 1:**
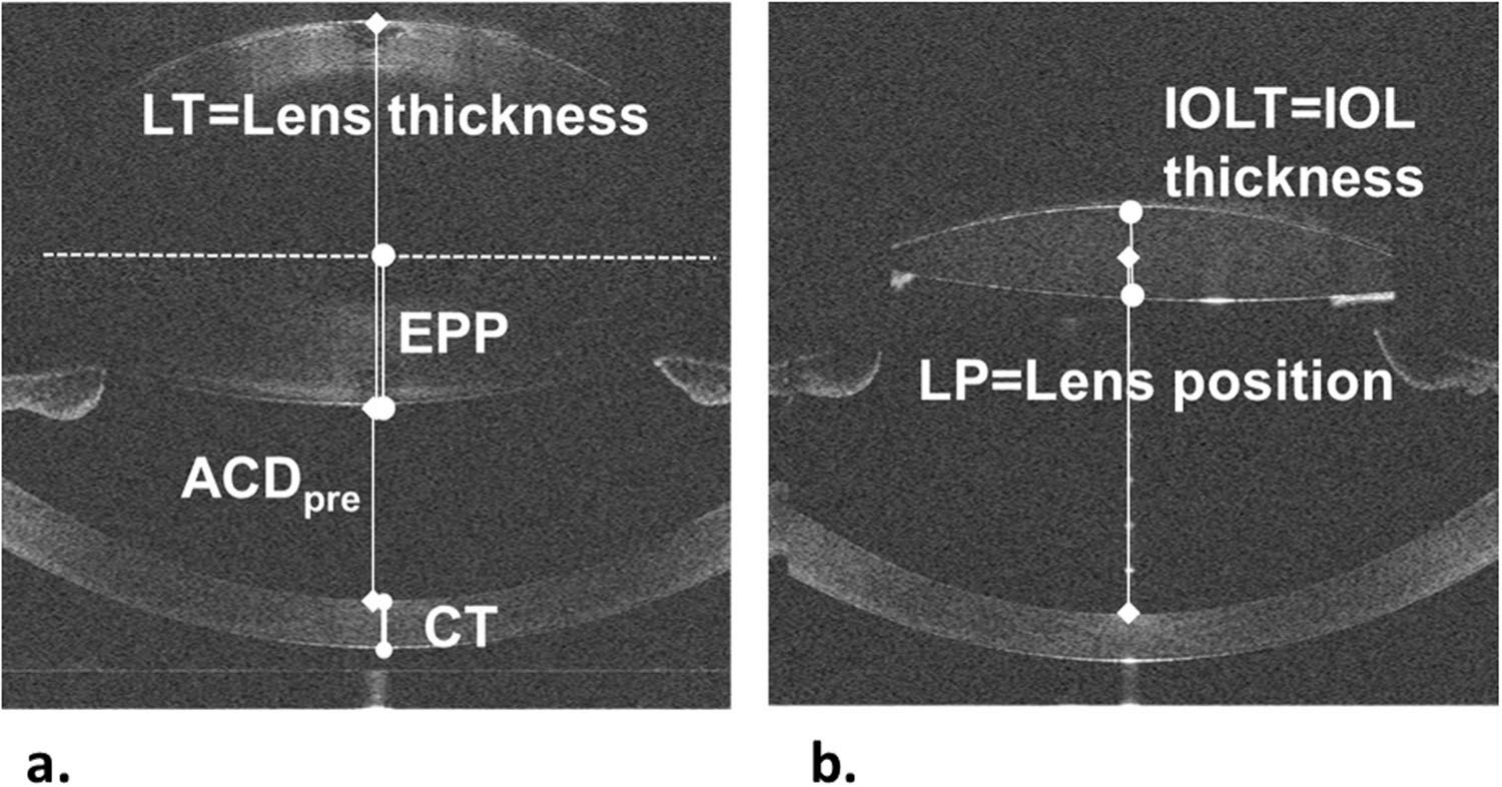
Raw optical coherence tomography (OCT) images for subject S#1 OD, including the definition of some biometric parameters. (**a**) Preoperative measurement. (**b**) Postoperative measurement. ACD_pre_ = anterior chamber depth, EPP = equatorial plane position, CT = corneal thickness.

Figure 1 shows examples of raw OCT central B-scans for subject S#1 OD for preoperative (Fig. 1a) and postoperative (Fig. 1b) measurements. B-scans taken at the different foci have been manually merged for representation purposes. Automatic registration is performed in 3-D using the iris as a reference, as explained below.

### 3-Dimensional Eye Model Construction

In previous works^22,26,27^ we described the automatic construction of accurate 3-D eye models from OCT images, which involves three steps: (1) automatic surfaces detection and segmentation, (2) registration, and (3) distortion correction. The custom-developed segmentation approaches are described in detail in previous work^22,26,27^. Specific routines have been designed in this study for the automatic detection of the IOL, including new segmentation functions. As in previous work, the automatically detected iris is used as a reference for image registration^26^. Due to thicker crystalline lenses in some older eyes –which prevented the iris to be captured in the posterior lens images- the specular reflex of the anterior lens was used for registration in selected cases. Fan and optical distortion correction algorithms were applied on the registered volumes by using 3-D ray tracing routines^19^. The corneal and aqueous humor group refractive indexes were taken as 1.385^28^, and 1.345, respectively, and the crystalline lens refractive index was obtained from the age-dependent expression derived by Uhlhorn *et al*.^29^. The Asphina IOL refractive index was 1.460^21^.

### Entire Lens Shape Estimation

In a recently published paper^23^ we proposed a method to estimate the entire lens geometry from the information of the lens visible through the pupil in OCT images. The entire crystalline lens is described by means of a parametric model with two parameters that were trained and validated using 27 *ex vivo* lenses, in which the information of the full lens was available. Using the trained parametric model and the information within the pupil, very realistic lenses are constructed *in vivo* with a small estimation error. This method was applied to study the changes of lens full shape parameters with accommodation^27^, in older patients with cataract^23^, and in animal models of myopia^30^.

**Figure 2:**
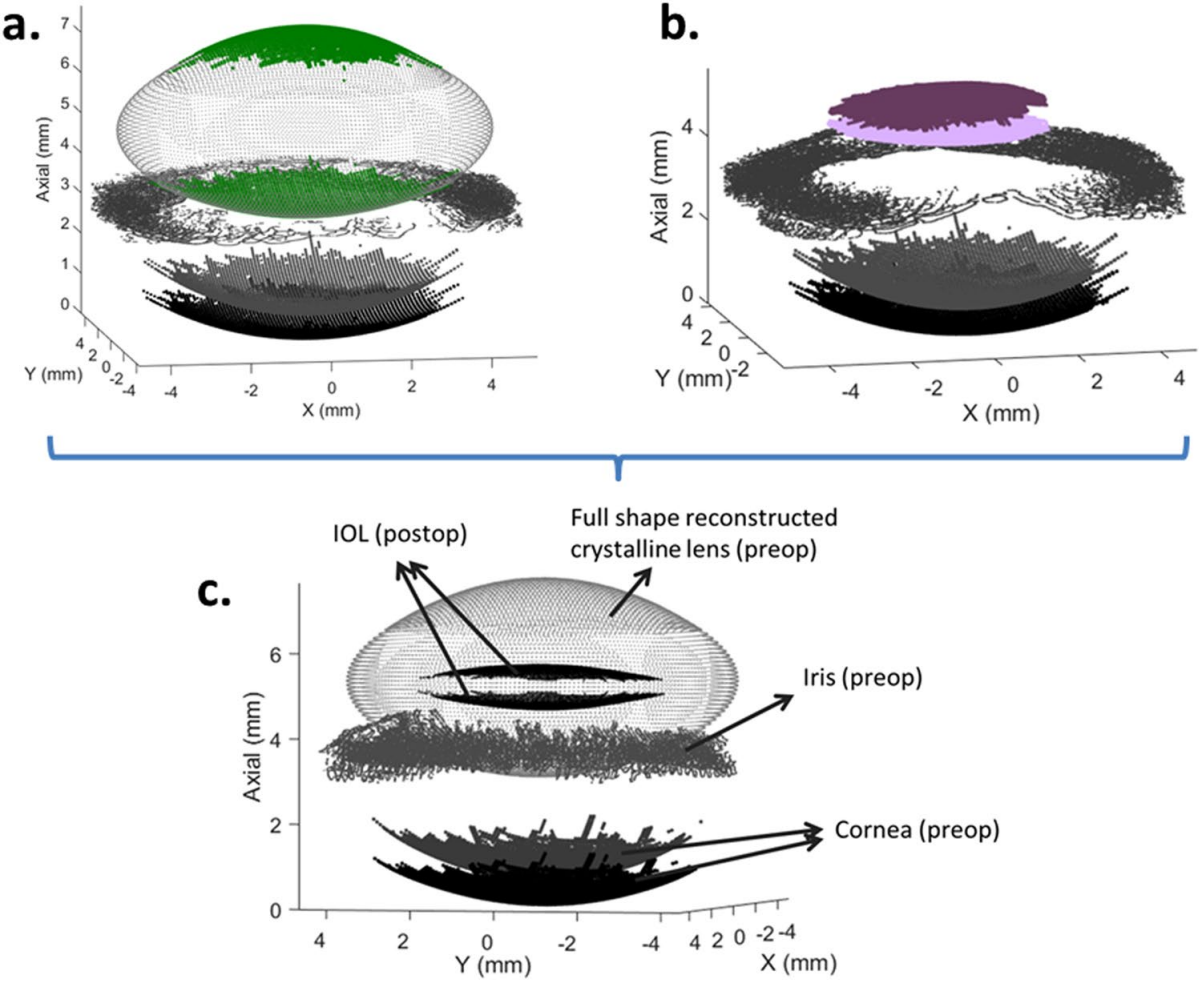
3-D models for S#1 (OD). (**a**) Preoperative measurement, including the part of the crystalline lens visible through the pupil (green) and its full shape estimation. (**b**) Postoperative measurement, including the intraocular lens (IOL) in purple. (**c**) Both models superimposed. IOL = intraocular lens.

Figure 2 shows constructed 3-D models from the preoperative measurement (Fig. 2a), including the part of the crystalline lens visible throughout the pupil (green) and its full shape estimation, as well as the postoperative measurement (Fig. 2b), with the IOL in purple for S#1 (OD). Both models superimposed are also shown (Fig. 2c).

### Quantification

Once the 3-D models were constructed, various biometric parameters were quantified. From the preoperative measurements, we obtained: (1) anterior chamber depth (ACD_pre_), defined as the distance between posterior cornea apex and anterior lens apex; (2) corneal thickness (CT); (3) lens thickness (LT); (4) crystalline lens anterior and posterior radius of curvature (RAL, RPL); (5) lens equatorial diameter (DIA); (6) lens equatorial plane position (EPP); (7) lens surface area (LSA); and (8) lens volume (VOL). From the postoperative measurements: (9) ACD_post_, defined as the distance from the posterior cornea to the IOL apex; (10) IOL thickness (IOLT); and (11) actual IOL position (LP), defined as the distance from the posterior cornea to the middle of the IOL: $LP=AC{D}_{post}+IOLT/2.$ Figure 1 illustrates the definition of some of these parameters. In particular, the EPP is the 3-D distance between the anterior lens apex and the position of the equatorial plane. More details of the different parameters definition are given in previous publications^23,27^. The crystalline lens anterior and posterior radii of curvature were calculated, after 6th order Zernike approximation and tilt removal of the lens, by fitting the best sphere in a 5-mm of diameter optical zone with respect to their apexes.

The analyzed crystalline lens parameters combinations included: (1) LSA/VOL ratio, which for a given volume reaches its minimum when the crystalline lens shape approaches a sphere, and (2) EPP/LT ratio, which gives the equator position in relation with the lens thickness.

### ELP and Estimation Error

We obtained the ELP as a function of the preoperative geometry of the anterior segment, including the full shape of the lens. In addition to the proposed ELP estimation, the ELP was also obtained from state-of-the-art methods, namely:

- Using the C constant for the corresponding lens (Asphina 409) recently proposed by Olsen and Hoffmann:^17^ 0.39.
- Using the C constant proposed by Olsen and Hoffmann optimized with our data set: 0.397.
- Using the ELP from the SRK/T formula^9^ using the A-constant recommended by the manufacturer: 118.3.
- Using the ELP from the SRK/T formula optimizing the A constant with our data set: A_opt_ = 118.5.
- Using the intersection approach, that estimates the ELP from the intersection of the two parametric surfaces that best fit the anterior and posterior crystalline lens surfaces within the pupil^18^.

The estimation error (EE) was calculated as the difference between the prediction using the different methods mentioned above and the actual lens position (LP, obtained from the postoperative measurements), i.e., *estimation error (EE)* = *estimated lens position (ELP)* − *measured lens position (LP)*.

Mean arithmetic EE (ME) across the 12 eyes and standard deviation of the ME were calculated. A positive value of ME indicates that the method predicts a position further from the anterior lens than the actual position of the lens, and vice versa for a negative value. Mean of the *absolute* value of the EE (MAE) and standard deviation of the MAE were also calculated.

The optimization of the C and A constants was done by finding those values that minimized the MAE.

### Postoperative Refraction Estimates and Error

To evaluate the improvements in terms of postoperative refraction estimation, the actual measured postoperative refraction (spherical equivalent, SE, Table 1) was compared in retrospect with the expected (predicted) refraction obtained with the SRK/T IOL power calculation formula, using the actual IOL power implanted and the different estimations of the IOL position (ELP). Thus, the refractive error (RE) is defined as: *RE* = *Measured_postoperative_refraction-estimated_refraction*.

The mean arithmetic RE (MRE) across the 12 eyes and the standard deviation of the RE were calculated. A positive value of MRE indicates that the method predicts a refraction lower than the postoperative measured refraction, and vice versa for a negative value. The mean of the *absolute* values of the RE (MARE) and standard deviation of the MARE across eyes were also calculated.

### Data Analysis

For all analyses, statistical significance was defined as a p-value lower than 0.05.

The normality of the data distribution was assessed for all the features and for the estimation errors with the Shapiro-Wilk test. When the data were not normally distributed, a square-root transformation was applied and the test was conducted over the transformed variable.

Linear regression analysis between pair of geometrical parameters of the crystalline lens and the lens position was performed, obtaining the Pearson correlation coefficient (r) and the p-value for testing the hypothesis of no correlation. Normality of the residuals was tested. Autocorrelation of the residuals was also tested with the Durbin-Watson test.

To asses if the ME and MRE produced by various methods were significantly different to zero, 1-sample t-test were used.

To assess if the MAE and the MARE produced by state-of-the-art methods were significantly different from the proposed, linear mixed-effects models (that handle repeated measures allowing an unequal number of repetitions) were used. As fixed effect, we entered methods into the model. As random effects, we had subjects, and as repeated measurements methods*eye.

The Bonferroni correction was applied for multiple tests. The SPSS 24.0 for Windows (SPSS Inc., Chicago, IL) was used for statistical analysis.

### Data availability

The datasets generated and/or analysed during the current study are available in the “Estimation-of-Intraocular-Lens-Position-from-Full-Crystalline-Lens-Geometry” repository, [https://github.com/EduardoMartinezEnriquez/Estimation-of-Intraocular-Lens-Position-from-Full-Crystalline-Lens-Geometry].

## Results

### Full Shape Crystalline Lens Geometry

Table 2 shows crystalline lens biometric parameters for each patient (mean ± standard deviation across measurements).

**Table 2:** Full shape of the crystalline lens biometric parameters for each patient (mean ± standard deviation across measurements).

	LT (mm)	RAL (mm)	RPL (mm)	VOL (mm^3^)	DIA (mm)	LSA (mm^2^)	EPP (mm)	EPP/LT	LSA/VOL (mm^−1^)
S#1 (OD)	4.62 ± 0.005	7.14 ± 0.21	5.57 ± 0.07	178 ± 3.7	8.83 ± 0.12	165 ± 3.7	2.16 ± 0.016	0.467	0.927
S#2 (OS)	4.35 ± 0.005	8.94 ± 0.49	5.80 ± 0.14	177 ± 5.0	9.00 ± 0.13	168 ± 4.1	1.92 ± 0.025	0.441	0.949
S#3 (OD)	5.67 ± 0.001	7.77 ± 0.18	5.46 ± 0.20	264 ± 2.5	9.51 ± 0.10	209 ± 1.0	2.63 ± 0.003	0.463	0.792
S#3 (OS)	5.61 ± 0.002	7.62 ± 0.06	5.05 ± 0.02	246 ± 1.6	9.36 ± 0.05	198 ± 1.0	2.57 ± 0.007	0.457	0.805
S#4 (OD)	4.79 ± 0.019	9.70 ± 0.21	5.84 ± 0.12	218 ± 0.4	9.54 ± 0.03	191 ± 0.8	2.10 ± 0.020	0.438	0.876
S#4 (OS)	4.75 ± 0.01	9.53 ± 0.19	5.90 ± 0.03	224 ± 2.5	9.77 ± 0.08	195 ± 1.5	2.14 ± 0.021	0.450	0.871
S#5 (OD)	4.41 ± 0.003	9.15 ± 0.56	5.41 ± 0.17	176 ± 5.1	8.92 ± 0.17	166 ± 4.2	1.94 ± 0.041	0.440	0.943
S#5 (OS)	4.55 ± 0.003	8.75 ± 0.23	5.88 ± 0.002	199 ± 0.4	9.26 ± 0.05	182 ± 1.3	2.05 ± 0.004	0.451	0.915
S#6 (OD)	4.18 ± 0.004	11.70 ± 0.63	6.54 ± 0.23	193 ± 4.5	9.60 ± 0.11	185 ± 3.5	1.84 ± 0.026	0.442	0.958
S#6 (OS)	4.17 ± 0.001	11.25 ± 0.70	6.37 ± 0.17	186 ± 5.0	9.43 ± 0.15	178 ± 1.8	1.84 ± 0.002	0.440	0.957
S#7 (OD)	5.23 ± 0.005	8.75 ± 0.06	5.43 ± 0.02	237 ± 4.2	9.60 ± 0.03	198 ± 2.2	2.32 ± 0.001	0.444	0.835
S#7 (OS)	4.78 ± 0.005	8.45 ± 0.29	5.74 ± 0.12	197 ± 4.4	9.09 ± 0.10	176 ± 3.2	2.20 ± 0.008	0.459	0.893

LT = lens thickness; RAL = crystalline lens anterior radius of curvature; RPL = crystalline lens posterior radius of curvature; VOL = lens volume; DIA = lens equatorial diameter; LSA = lens surface area; EPP = equatorial plane position.

**Figure 3:**
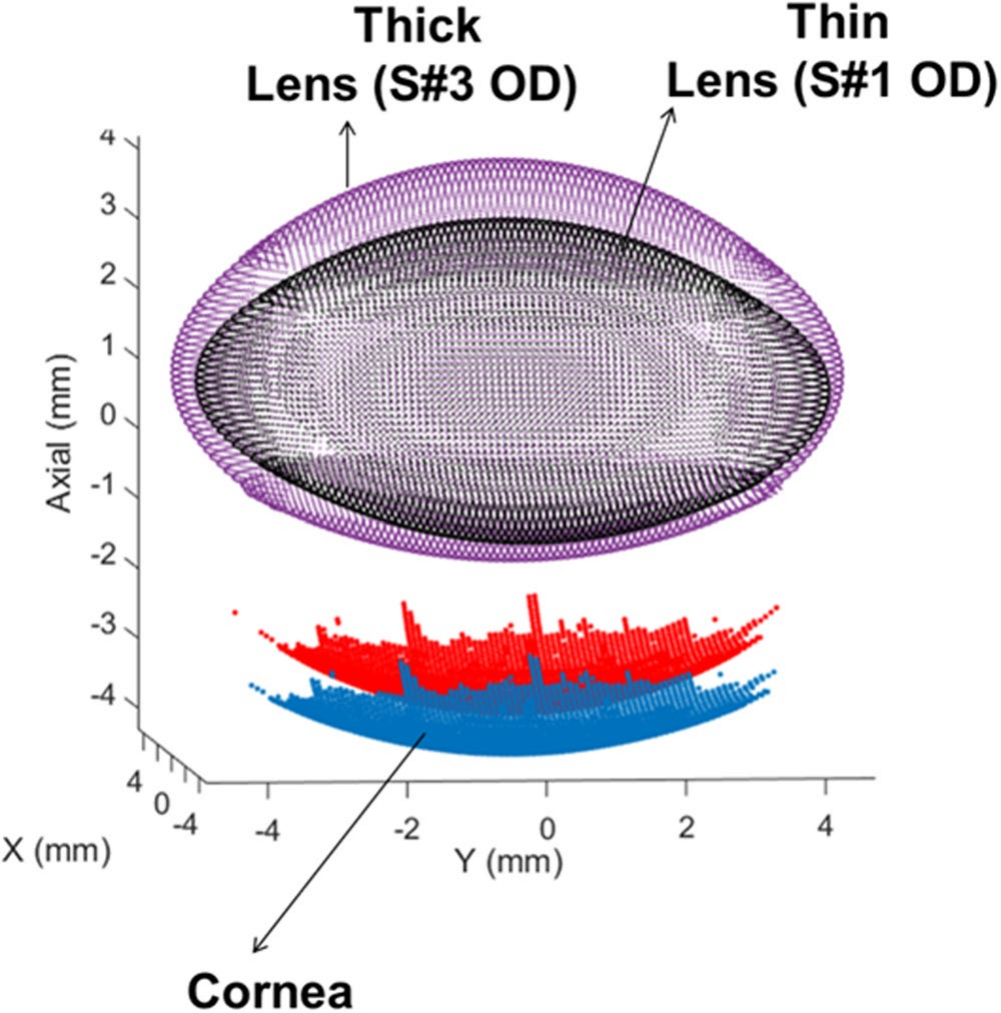
Graphical comparison of 3-D models for a thin lens (S#1 OD) and a thick lens (S#3 OD) using the real geometries obtained from the analysis. Cornea was used for registration.

We found a significant correlation between Lens Thickness (LT) and: Volume,VOL (r = 0.905, p = 5.1·10^−5^); Lens Surface Area, LSA (r = 0.76, p = 0.004); Equatorial Plane Position, EPP (r = 0.98, p = 2.5·10^−9^); Radius of the Anterior Lens, RAL (r = −0.66, p = 0.019); Radius of the Posterior Lens, RPL (r = −0.75, p = 0.0046), LSA/VOL (r = −0.98, p = 1.5·10^−8^); Preoperative Anterior Chamber Depth, ACDpre (r = −0.83, p = 8.5·10^−4^) and age (0.69, p = 0.013). The correlation was not significant with Lens Diameter, DIA (r = 0.27, p = 0.39) or EPP/LT ratio (r = 0.53, p = 0.07). Higher LT was associated with higher VOL and LSA, lower RAL and RPL (i.e., more curved surfaces), lower LSA/VOL ratio (more rounded lenses), lower ACD_pre_, and higher EPP/LT (the equatorial plane position tends to be closer to LT/2). Figure 3 shows a graphical comparison of a thick crystalline lens (S#3 OD, purple) and a thin crystalline lens (S#1 OD, black), using the real geometries obtained from the analysis. In this particular example, the cornea, captured in both models, was used for registration.

### Proposed ELP Estimation from the Preoperative Full Shape of the Lens Geometry

**Figure 4:**
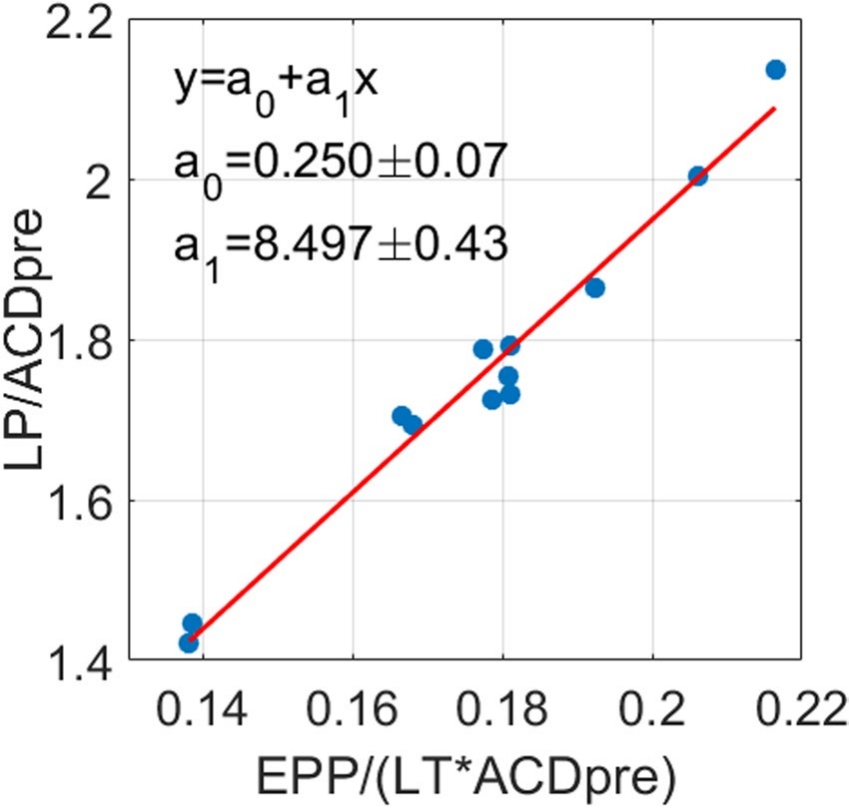
Linear regression between EPP/(LT·ACD_pre_) and ELP/ACD_pre_. r = 0.98, p = 3.6·10^−9^; $\frac{{\bf{LP}}}{{\bf{AC}}{{\bf{D}}}_{{\bf{pre}}}}={\bf{0}}{\bf{.250}}{\boldsymbol{(}}\,{\boldsymbol{\pm }}\,{\bf{0}}{\bf{.07}}{\boldsymbol{)}}$ + ${\bf{8}}{\bf{.497}}{\boldsymbol{(}}\,{\boldsymbol{\pm }}\,{\bf{0}}{\bf{.43}}{\boldsymbol{)}}\cdot \frac{{\bf{E}}{\bf{P}}{\bf{P}}}{{\boldsymbol{(}}{\bf{L}}{\bf{T}}{\boldsymbol{\cdot }}{\bf{A}}{\bf{C}}{{\bf{D}}}_{{\bf{p}}{\bf{r}}{\bf{e}}}{\boldsymbol{)}}}{\boldsymbol{.}}$ EPP = equatorial plane position, LT = lens thickness, ACD_pre_ = anterior chamber depth preoperative, ELP = estimated lens position, LP = lens position.

ELP was estimated using the EPP calculated from the full shape of the lens. Linear regressions between the available data revealed that the highest correlation occurred between the ratio EPP/(LT·ACD_pre_) and the ratio ELP/ACD_pre_ (r = 0.98, p = 3.6·10^−9^). Figure 4 shows a scatter plot and the regression line given by

$$\frac{{\bf{L}}{\bf{P}}}{{\bf{A}}{\bf{C}}{{\bf{D}}}_{{\boldsymbol{p}}{\boldsymbol{r}}{\boldsymbol{e}}}}={\bf{0.250}}(\,\pm \,{\bf{0.07}})+{\bf{8.497}}(\,\pm \,{\bf{0.43}})\cdot \frac{{\bf{E}}{\bf{P}}{\bf{P}}}{({\bf{L}}{\bf{T}}\cdot {\bf{A}}{\bf{C}}{{\bf{D}}}_{{\boldsymbol{p}}{\boldsymbol{r}}{\boldsymbol{e}}})}.$$

Reordering the equation of the best linear fitting (solving for LP), the proposed estimation linearly relates the LP with the EPP/LT ratio and the ACD preoperative (ACD_pre_) as follows:

1 $${\bf{ELP}}={\bf{8.497}}\cdot \frac{{\bf{EPP}}}{{\bf{LT}}}+{\bf{0.250}}\cdot {\bf{AC}}{{\bf{D}}}_{{\bf{pre}}}{\boldsymbol{,}}$$

where LT is the measured lens thickness and ACD_pre_ is the preoperative anterior chamber depth (Fig. 1). As expected, the lens position increases with the EPP/LT ratio (i.e., as the equatorial plane position is closer to the half of the lens thickness) and with the ACD_pre_.

Alternatively, another relationship with high correlation was found between the ratios ELP/LT and LSA/VOL (r = 0.97, p = 1.4·10^−7^), leading to the formula:

2 $${\bf{E}}{\bf{L}}{\bf{P}}={\bf{1.72}}\cdot {\bf{L}}{\bf{T}}\cdot \frac{{\bf{L}}{\bf{S}}{\bf{A}}}{{\bf{V}}{\bf{O}}{\bf{L}}}-{\bf{0.59}}\cdot {\bf{L}}{\bf{T}}.$$

In the following we will employ equation (1) as the proposed method to obtain the ELP.

### ELP Estimation Error

Table 3 shows the ME, MAE, EE range and p value of the statistical tests. The ME was close to zero for all methods, except for the SRK/T, in which the ME was significantly different to zero. For the proposed method, the standard deviation of the ME (81 µm) and the EE range (−102 to 144 µm) were smaller than those obtained by the other methods.

**Table 3:** ELP estimation error: mean arithmetic error (ME), mean absolute error (MAE), estimation error (EE) range, and p values of the statistical tests performed, for the different methods.

Method	ME ± SD (µm)	P value (t-test)	MAE ± SD (µm)	EE range (µm)	P value (multiple test, Bonferroni correction)
Proposed	0 ± 81	1	65 ± 45	−102 to 144	—
Olsen Constant	−26 ± 186	0.64	158 ± 91*	−297 to 289	0.02
Olsen C optimized	1 ± 181	0.97	145 ± 101	−263 to 312	0.34
SRK/T	−187 ± 211^Ɨ^	0.01	238 ± 145*	−501 to 125	0.02
SRK/T A optimized	0 ± 182	0.99	151 ± 91	−350 to 250	0.65
Intersection approach	−79 ± 201	0.20	177 ± 114*	−352 to 380	0.04

ME = Mean arithmetic estimation error; MAE = Mean absolute estimation error; SD = Standard deviation; EE = Estimation error.

^Ɨ^ME significantly different from zero (t-test).

^*^MAE significantly different than the proposed method (with Bonferroni correction).

The MAE with the proposed method was statistically significantly lower (multiple comparisons, Bonferroni correction) than with the C constant by Olsen, the SRKT and the intersection approach. The EE for every eye when using the proposed method, the optimized C constant, the optimized SRK/T, and the intersection approach can be found as Supplementary Fig. S1.

### Postoperative Refraction Estimation

Table 4 shows the MRE, MARE, RE range and p value of the statistical tests. The MRE was not significantly different to zero for any of the compared methods. For the proposed method, the standard deviation of the MRE (0.37 D) was smaller than for the other methods. The RE range (−1.08 to 0.33 D) was also smaller with the proposed method than for the other methods except for the intersection approach (−0.83 to 0.53 D). The MARE was not significantly different across the different methods (multiple comparisons, Bonferroni correction).

**Table 4:** Postoperative refraction estimation: mean arithmetic refractive error (MRE), mean absolute refractive error (MARE), refractive error (RE) range, and p values of the statistical tests performed, for the different methods.

Method	MRE ± SD (D)	P value (t-test)	MARE ± SD (D)	RE range (D)	P value (multiple test, Bonferroni correction)
Proposed	−0.09 ± 0.37	0.41	0.24 ± 0.29	−1.08 to 0.33	—
Olsen Constant	−0.04 ± 0.46	0.75	0.36 ± 0.27	−0.95 to 0.63	1
Olsen C optimized	−0.08 ± 0.46	0.54	0.37 ± 0.26	−1.00 to 0.58	1
SRK/T	0.20 ± 0.53	0.23	0.47 ± 0.28	−0.73 to 0.93	0.16
SRK/T A optimized	−0.08 ± 0.53	0.61	0.41 ± 0.31	−1.20 to 0.62	1
Intersection approach	0.03 ± 0.44	0.81	0.37 ± 0.23	−0.83 to 0.53	0.12

MRE = mean arithmetic refractive error; MARE = mean absolute refractive error; SD = Standard deviation;

EE = Estimation error; D = diopters.

^Ɨ^RE significantly different from zero (t-test).

*MARE significantly different than the proposed method (with Bonferroni correction).

**Figure 5:**
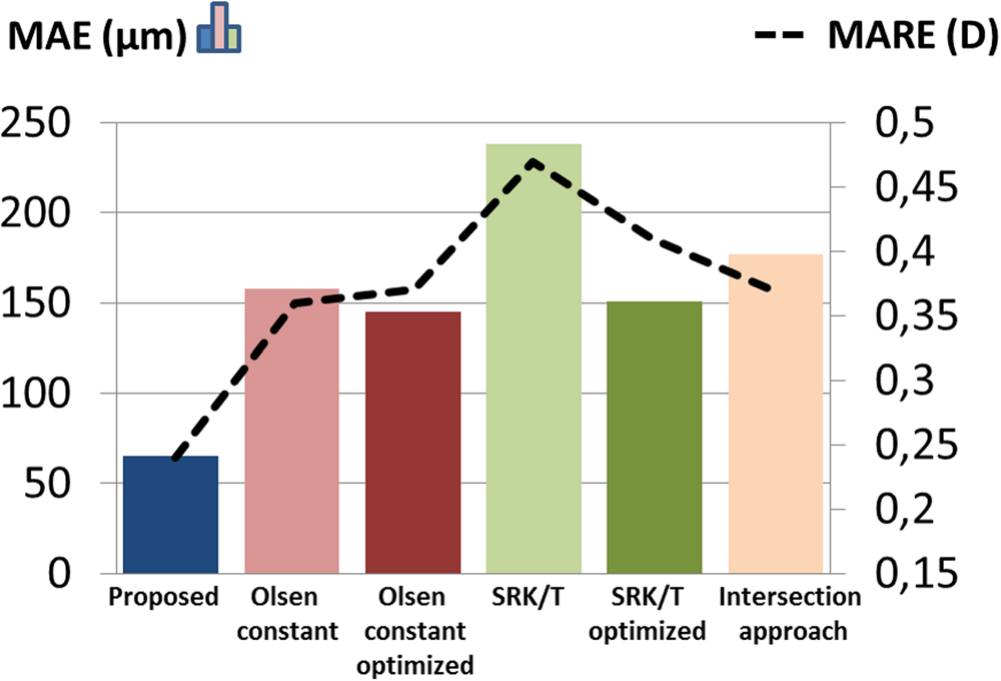
Mean of the *absolute* value of the estimation error (MAE, color bars) and of the refractive error (MARE, black dotted line) across the 12 eyes, for the compared estimation methods.

Figure 5 shows the MAE (color bars) and the MARE (black dotted line) for all the compared estimation methods.

The RE for every eye when using the proposed method, the optimized C constant, the optimized SRK/T, and the intersection approach can be found as Supplementary Fig. S2.

## Discussion

We proposed a new method to estimate the postoperative IOL position (ELP) from preoperative 3-D geometry of the crystalline lens. A more accurate estimation of the IOL position leads to improved formulas for IOL power estimation in which ELP is a primary source of error, and opens new avenues to the development of new strategies for IOL power estimation based on ray tracing.

The mean absolute estimation error (MAE) of the LP obtained in this study with the proposed method was >2 times lower than the error using the C constant by Olsen (even if the constant was optimized with our data set) or the SRK/T with the A constant optimized, and around 3 times lower than using the ELP estimate of the SRK/T or the intersection approach (Table 3, Fig. 5). These differences were statistically significant (multiple comparisons, Bonferroni correction) between the proposed method and the Olsen constant, the SRK/T, and the intersection approach.

Optimizing the C and the A constants reduced the error by 1.09 and 1.57 times with respect to the constants recommended by Olsen and the manufacturer respectively.

The deviation in the ELP estimation error across patients was lower with the proposed formula than using other methods (±81 µm with the proposed vs ±181 to ±211 µm with the other methods), and the estimation error range was also lower (−102 to 144 µm with the proposed vs −263 to 312 and −352 to 380 µm with other methods), indicating that the proposed method provides a more individualized account of the lens position than compared methods in the studied sample.

Figure 5 shows that the MAE and the MARE were well correlated (r = 0.94, p = 0.005), indicating that an improvement in the ELP estimation should result in an improvement in the postoperative refraction. Comparing the absolute RE obtained subject by subject (see Supplementary Fig. S2) with the proposed method and with all the other methods (12 eyes*5 methods = 60 data), the improvement in refraction produced by the proposed approach was greater than 0.15 D in 32 cases, greater than 0.25 D in 18 cases and greater than 0.5 D in 3 cases; the estimation was >0.15 D worse in 4 cases and >0.25 D worse just in 2 cases. As with the ELP, the standard deviation of the refraction estimation error across eyes using the proposed ELP (±0.37 D) was lower than with the other methods (from ±0.44 D to ±0.53 D), indicating a more customized performance for the different subjects of the study.

Although improvement in RE was found in most eyes, MARE differences between the proposed method and the compared methods were not proven to be significantly different (multiple comparisons, Bonferroni correction), likely due to the high data dispersion and the low number of eyes (N = 12). Clinical relevance may be achieved when combining this methodology with further methods that use additional anatomical information of the patient (i.e. patient-based custom eye models using anterior and posterior corneal topography, IOL geometry, and ray tracing). A larger sample population may be required to generalize the formula for clinical use.

Note that the proposed estimations were compared to those provided by SRK/T because it is one of the most commonly used formulas in clinic and because it was the method used by the surgeon for IOL power selection in all the patients in the present work. We also compared with Olsen’s C constant and the intersection approach because, as similarly to our proposed method, these methods use crystalline lens parameters to estimate the ELP. There are several anterior segment OCT instruments clinically available (corneal topographers; 2-D optical biometers and OCT units to guide femtosecond laser cataract surgery) that could be envisioned to be modified and coupled with the quantification tools described in this study to be used in the optimization of the selection of IOLs.

In a clinical application, typical IOLs are sold in 0.5 D increments. Comparing with SRK/T, the proposed approach would result in a different preoperative selection of IOL power in 5 of the 12 eyes, following the same selection criteria by the same surgeon. Specifically, IOL power would change in 0.5 D for 2 eyes and 1 D for 3 eyes.

Advanced cataracts may complicate measuring the posterior lens surface or the lens thickness. We did not find any difficulty to capture the posterior lens in cataracts of grade up to N6 (over N10) in our study. Besides, recent works show cataract crystalline lens images up to grade 6 in LOCS III grading^31^ and excellent results (no failures in a sample of 188 eyes of different cataract grades) in axial measurements (ACD, AL) in the OCT based IOLMaster 700^32^. The changes in the refractive index with the cataract grade are not well known. We did not find any relation between the grade of the cataract and the ELP estimation error in our study. Although changes in the refractive index may affect the estimation of the EPP, ELP estimation formula (1) is quite robust because it does not directly depend on the considered refractive index of the crystalline lens (the ratio EPP/LT does not depend on the refractive index).

The use of 3-D full shape information of the crystalline lens goes beyond the state-of-the-art, as current approaches use, at most, lens thickness (1-D) or lens cross-sectional data of the central part. In this study, we proposed a specific formula that estimates the ELP from 3-D full shape of the crystalline lens, in particular the equatorial plane position (EPP), the thickness of the crystalline lens (LT) and the ACD_pre_. Nevertheless, other relationships (such as that shown in equation (2)), can be obtained that use other lens 3-D parameters (i.e. LSA and VOL) to derive ELP. We did not attempt to use a larger number of features in the equation (1) for the ELP estimation to avoid over-fitting, given the small data set (12 eyes), and prevent an unfair comparison with other methods. However, the fact that we obtained lower MAE over other ELP approaches also optimized with the same dataset (such as the C-Constant formula of Olsen or the A constant in the SRK/T) suggests that it is the use of a more comprehensive description of the crystalline lens, and not the optimization to the specific eyes, the reason behind the improvement.

This study demonstrates the potential opened by image-based 3-D quantification of the preoperative crystalline lens to improve cataract surgery. The retrieved formulas (equations (1)-(2)) or even other alternatives can be generalized using a larger clinical population. The availability of larger dataset will allow to use powerful tools to systematically retrieve the best set of features^33^ (i.e. those most informative, such us, lens geometrical parameters, age^34^, preoperative refraction, …) as well as a mathematical expression (N-dimensional, linear or non-linear) depending on these features to estimate the ELP.

Improving the generalization will allow obtaining formulas that more accurately estimate the IOL position of outliers (e.g., extreme axial lengths^35^) or after refractive surgery, which creates difficulties in IOL power calculation^36,37^.

The refraction predictions have been obtained using the proposed ELP in the SRK/T formula for IOL power selection. However, our ELP estimation can be used in different current IOL power calculation formulas to improve the refraction prediction and thus the IOL selection.

While most current formulas assume some simplifications (e.g., the posterior cornea radius of curvature is not considered, assumption of a keratometric index), ray tracing–based IOL power estimations are not subject to these assumptions. Computer eye models for ray tracing customized to the patient’s anatomy^21^ can be constructed using all inputs from quantitative 3-D OCT (anterior and posterior corneal topography, 3-D biometry, IOL tilt and decentration and the proposed ELP based on the full shape of the crystalline lens), further increasing the accuracy of the IOL power selection, particularly in non-standard cases such us irregular corneas or post-refractive surgery eyes.

In the current study we obtained all anterior segment parameters from the same spectral OCT system, and axial length from a commercial low coherence interferometry system. However, the larger range of swept source OCT systems will allow acquiring all the information from a single instrument.

It should be noted that the proposed formula has been obtained for the specific implanted IOL (Asphina 409 M). Different constants in the formula are likely to result from different IOL platforms^17^.

The proposed estimate of ELP relies on a reliable estimation of the crystalline lens parameters. We found high repeatability in the parameter estimation from repeated measurements on the same eye: the mean across eyes of the standard deviation of repeated measurements was 0.014 mm for EPP, 3.30 mm^3^ for VOL, 3.24 mm^2^ for LSA, 0.092 mm for DIA, and 0.005 mm for LT.

The technique for full lens shape estimation has been validated in eyes *ex vivo*, and further supported *in vivo* in young and presbyopic eyes^23,27^. In previous studies we found that the assumption of an equivalent homogeneous refractive index in the lens (instead of the GRIN) resulted in <1.5% inaccuracies in VOL, and the effect of OCT lateral sampling, axial resolution, and registration errors resulted in <1% inaccuracies in EPP, VOL and DIA, which translates into <1% in the ELP. Furthermore, the application in older lenses reduces potential criticisms of the technique which may play some role in younger lenses. The training set of *ex vivo* lenses used to develop the parametric full lens model mainly corresponded to nearly presbyopic or presbyopic eyes, where one expects minimal changes with accommodation and thus *ex vivo* and *in vivo* lenses are expected to have approximately the same shape. On the other hand, GRIN is expected to be much flatter in older lenses, thus minimizing the error related to the assumption of constant index of refraction in the calculations of the current paper.

Postoperative measurements were obtained 2 to 4 months after surgery. Long-term (1 day to 1 year) changes in refractive outcomes in single-piece IOLs (as the IOL of the present study) due to IOL position shifts have been reported to be of negligible clinical relevance^38,39^. Further analysis will be needed to potentially incorporate IOL postoperative shifts in other types of IOLs (e.g., multipiece IOLs).

Our results show an improvement in the estimation of the lens position over approaches based on limited anatomical information (e.g., only the radius of curvature of anterior cornea and the axial length of the eye in the SRK/T ELP). These results further improve over previous proposals which included for the first time lens data (e.g., Olsen constant or the intersection approach). Thus, knowledge of the full lens shape, and in particular EPP, is very valuable to estimate ELP.

In summary, 3-D OCT with image processing, distortion correction, and full-shape estimation of the crystalline lens algorithms allows accurate quantification of the anterior segment of the eye, which is very useful for improving the preoperative estimation of the postoperative IOL position (ELP).

Patient-specific eye models that include the information on lens volume, surface area, diameter, and equatorial plane position, open the possibility for development a new generation of IOL power estimation formulas.

## Electronic supplementary material

Supplementary material
