# Supplementary material for: Estimation of intraocular lens position from full crystalline lens geometry: towards a new generation of intraocular lens power calculation formulas
Source: Sci Rep. 2018 Jun 29;8:9829. doi: 10.1038/s41598-018-28272-6 (PMC6026180; doi:10.1038/s41598-018-28272-6)
Supplement: Supplementary file 1 — Supplementary material [file 41598_2018_28272_MOESM1_ESM.pdf]

Estimation of Intraocular lens position from full crystalline lens geometry: towards a new generation of intraocular lens power calculation formulas

Authors:

Eduardo Martinez-Enriquez,*[1] Pablo Pérez-Merino,[1] Sonia Durán-Poveda,[2] Ignacio Jiménez-Alfaro,[2] Susana Marcos.[1]

Affiliations:

[1] Instituto de Óptica "Daza de Valdés", Consejo Superior de Investigaciones Científicas (CSIC), C/Serrano, 121, 28006, Madrid, Spain.

[2] Fundación Jiménez Díaz, Madrid, Av. Reyes Católicos, 2, 28040 Madrid, Spain.

Corresponding author:

Name: Eduardo Martinez-Enriquez.

Address: Instituto de Óptica "Daza de Valdés", Consejo Superior de Investigaciones Científicas (CSIC), C/Serrano, 121, 28006, Madrid, Spain.

Phone number: +34 915 616 800

Fax number: +34 915 645 557

e-mail: eduardo.martinez@io.cfmac.csic.es

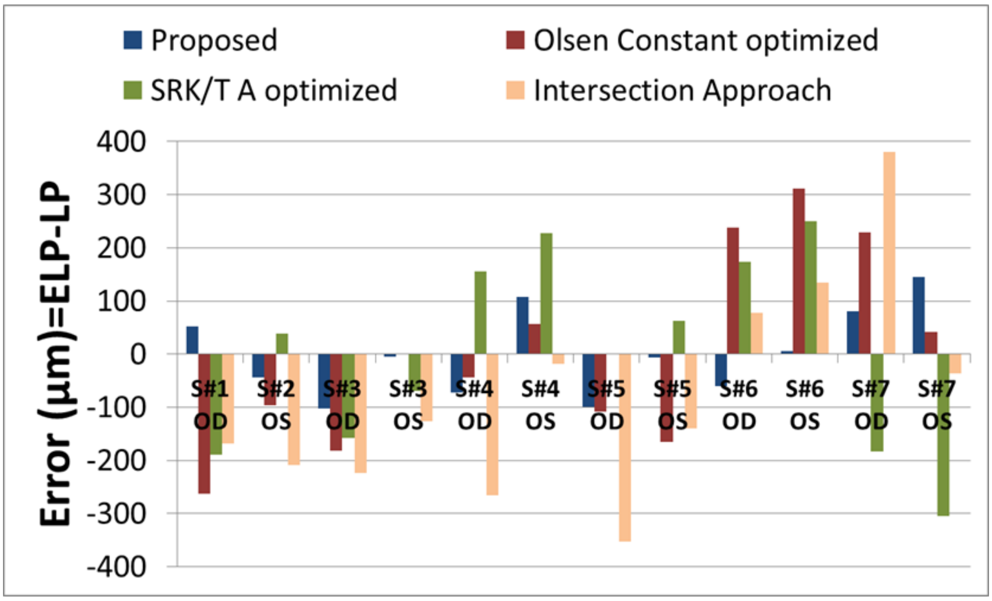

SUPPLEMENTARY FIGURE 1. Estimation error (EE) in all eyes, calculated as *estimation error (EE)=estimated lens position (ELP)- measured lens position (LP)*, for some of the compared estimation methods.

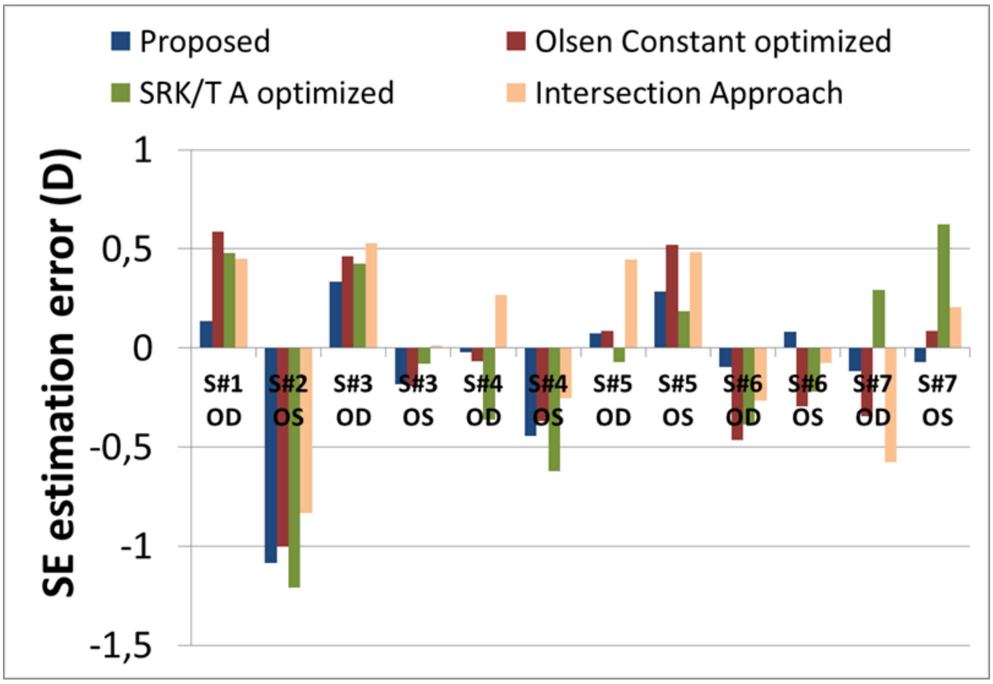

SUPPLEMENTARY FIGURE 2. Estimation error of the postoperative spherical equivalent (RE) for all eyes, calculated as *RE=Measured_postoperative_refraction-estimated_refraction*, for the proposed method, the optimized C constant and SRK/T, and the intersection approach lens position estimation (ELP) methods.
